# Tamoxifen associated to the conservative CKD treatment promoted additional antifibrotic effects on experimental hypertensive nephrosclerosis

**DOI:** 10.1038/s41598-023-39299-9

**Published:** 2023-08-26

**Authors:** Camilla Fanelli, Ana L. R. Francini, Giovanna A. Celestrino, Flávio Teles, Ana P. Barbosa, Paloma Noda, Leandro R. Iannuzzi, Cristiane R. Guzzo, Felipe M. Ornellas, Irene L. Noronha

**Affiliations:** 1 Laboratory of Cellular, Genetic, and Molecular Nephrology, Renal Division, University of São Paulo Medical School, São Paulo - SP, Brazil; 2 Faculty of Medicine, Federal University of Alagoas, Av. Lourival Melo Mota, S/N Tabuleiro do Martins, Maceió - AL, 57072-900 Brazil; 3 Institute of Biomedical Sciences, University of São Paulo, São Paulo - SP, Brazil

**Keywords:** Physiology, Drug discovery, Target identification, Target validation, Nephrology, Kidney diseases, Diseases, Kidney diseases, Chronic kidney disease, Nephrosclerosis, Renal fibrosis

## Abstract

CKD progression depends on the activation of an intricate set of hemodynamic and inflammatory mechanisms, promoting renal leukocyte infiltration, inflammation and fibrosis, leading to renal function loss. There are currently no specific drugs to detain renal fibrogenesis, which is a common end-point for different nephropathies. Clinical therapy for CKD is mostly based on the management of hypertension and proteinuria, partially achieved with renin–angiotensin–aldosterone system (RAAS) blockers, and the control of inflammation by immunosuppressive drugs. The aim of the present study was to verify if the administration of tamoxifen (TAM), an estrogen receptor modulator, clinically employed in the treatment of breast cancer and predicted to exert antifibrotic effects, would promote additional benefits when associated to a currently used therapeutic scheme for the conservative management of experimental CKD. Wistar rats underwent the NAME model of hypertensive nephrosclerosis, obtained by daily oral administration of a nitric oxide synthesis inhibitor, associated to dietary sodium overload. The therapeutic association of TAM to losartan (LOS), and mofetil mycophenolate (MMF) effectively reduced the severe hypertension, marked albuminuria and glomerular damage exhibited by NAME animals. Moreover, the association also succeeded in limiting renal inflammation in this model, and promoted further reduction of ECM interstitial accumulation and renal fibrosis, compared to the monotherapies. According to our results, the association of TAM to the currently used conservative treatment of CKD added significant antifibrotic effects both in vivo and in vitro, and may represent an alternative to slow the progression of chronic nephropathy.

## Introduction

The pathogenesis of chronic kidney disease (CKD) involves an intricate process of both hemodynamic and inflammatory mechanisms that leads to renal fibrosis and progressive loss of function. Glomerular and systemic hypertension, increased production of cytokines and growth factors, renal infiltration of inflammatory cells, inordinate fibroblast proliferation and transdifferentiation into myofibroblasts have been described in different human nephropathies and experimental CKD models^1–4^.

The overactivation of both systemic and intrarenal renin–angiotensin–aldosterone system (RAAS) contributes to the progression of CKD^5,6^. Once bound to its specific receptor (AT1), the active peptide Angiotensin II (AII) promotes renal and systemic vasoconstriction and tubular sodium conservation, leading to the elevation of blood pressure^1,2,6^. AII also exerts proinflammatory effects, once it stimulates cell proliferation, fibroblast activation and further accumulation of extracellular matrix (ECM). Actually, since the discovery of the angiotensin ii converting enzyme inhibitors (ACEi) and the AT1 receptor blockers (ARB), such as losartan (LOS), RAAS suppression remains to be the best option available to slow the progression of CKD, although this strategy does not fully halt the progression of renal fibrosis and loss of function^7–10^.

It is well known that inflammation and increased ECM production exert an important pathogenic role to the development and progression of CKD, regardless of its etiology. Accordingly, we have previously demonstrated that treatment with anti-inflammatory drugs such as mycophenolate mofetil (MMF), which promotes antiproliferative effects on T-cells, presented effective renoprotection in experimental CKD, in the hypertensive nephrosclerosis model obtained by the chronic inhibition of Nitric Oxide (NO) synthesis associated to a high sodium (HS) diet (NAME model), in the 5/6 nephrectomy (Nx) and in the streptozotocin-induced diabetes^11–13^. Moreover, Nx rats treated with an association of MMF + LOS presented significantly less severe CKD when compared to animals receiving the respective monotherapies^14^.

Considering that renal fibrosis with tissue scarring is the final common pathway of CKD, therapeutic interventions with antifibrotic drugs could represent an attractive choice of therapy to arrest fibrogenesis in progressive nephropathies. In this context, tamoxifen (TAM), an estrogen receptor modulator clinically used in the treatment of breast cancer, has been demonstrated to also be effective in treating abnormal healing disorders, such as retroperitoneal fibrosis, sclerosing encapsulated peritonitis, fibrosing mediastinitis, among other fibroproliferative conditions^15–18^.

Additionally, we have recently shown that TAM treatment prevented the development of glomerulosclerosis and interstitial expansion in rats submitted to NO inhibition, even having no effects on the marked hypertension characteristic of this model^19^.

Motivated by the positive responses achieved by each employed monotherapy in the treatment of progressive nephropathy associated to the NAME model of CKD, in the present study, we sought to verify if the administration of an association of LOS + MMF + TAM could promote additional renoprotection compared to the respective monotherapies, once the mechanisms of action of each drug are different and somehow complementary.

## Results

### LOS + MMF + TAM was safe and effective in reducing blood pressure and albuminuria

**Figure 1:**
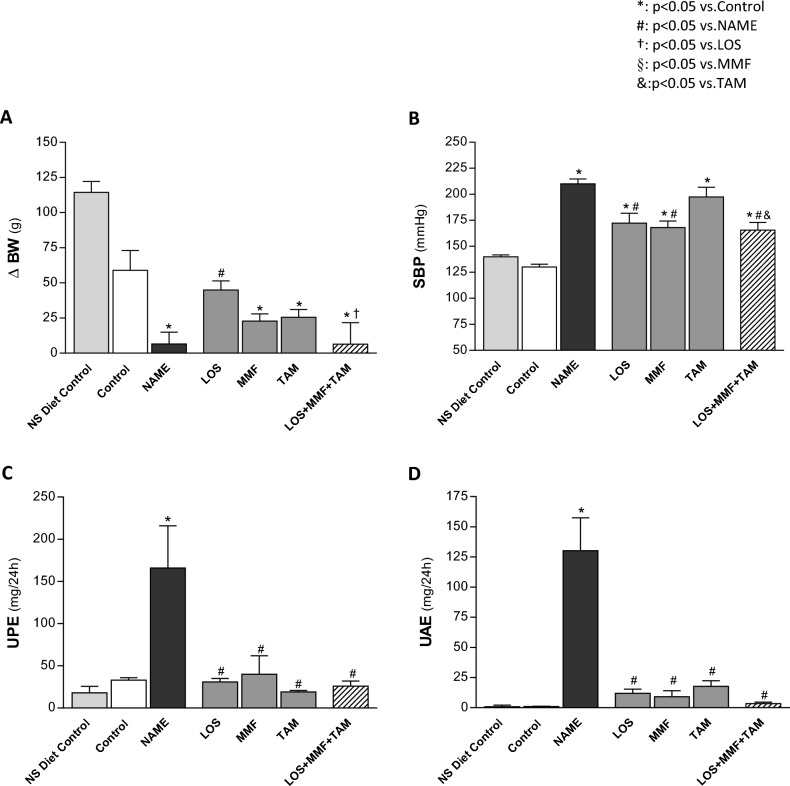
General parameters: bar graph representation of (**A**) delta of body weight (ΔBW, g), (**B**) systolic blood pressure (SBP, mmHg), (**C**) urinary protein excretion rate (UPE, mg/24 h) and (**D**) urinary albumin excretion rate in the NS diet control, control, NAME, LOS, MMF, TAM and LOS + MMF + TAM groups. Results are presented as mean ± SEM. *p < 0.05 vs. Control, #: p < 0.05 vs. NAME, †: p < 0.05 vs. LOS, §: p < 0.05 vs. MMF, &: p < 0.05 vs. TAM.

As can be seen in Fig. 1A, all Wistar rats fed with HS diet presented lower body weight gain (ΔBW) in 30 days, when compared to the animals which receive conventional normal-sodium (NS) diet during the same period (59 ± 14 vs. 115 ± 8 g). Corroborating previous findings, animals submitted to the chronic NO synthesis inhibition exhibited significantly lower ΔBW, even compared with the rats treated only with HS diet (8 ± 7 vs. 59 ± 14 g). Losartan was effective in normalizing this parameter; ΔBW of LOS group was 45 ± 7 g.

As expected, rats receiving L-NAME exhibited severe hypertension when compared to Control (210 ± 5 vs. 130 ± 3 mmHg). LOS or MMF as monotherapies, as well as the association of LOS + MMF + TAM promoted a partial reduction in the systolic blood pressure (SBP) levels (172 ± 9, 168 ± 6 and 166 ± 7 mmHg, respectively), as shown in Fig. 1B. NAME animals also developed marked proteinuria (UPE, 166 ± 50 vs. 33 ± 3 mg/24 h in the Control group) and albuminuria (UAE: 130 ± 27 vs. 1 ± 1 mg/24 h in the Control group), which were significantly reduced with all the employed therapies; LOS (UPE: 31 ± 4 and UAE: 12 ± 4 mg/24 h), MMF (UPE: 40 ± 22 and UAE: 9 ± 5 mg/24 h), TAM (UPE: 19 ± 2 and UAE: 18 ± 5 mg/24 h) and the association of LOS + MMF + TAM (UPE: 26 ± 6 and UAE: 3 ± 1 mg/24 h), as shown in Fig. 1C and D. It is worth mentioning that the treatment with combined LOS + MMF + TAM promoted the most prominent reduction in albuminuria, which reached final values closely similar to those observed in Control group.

**Figure 2:**
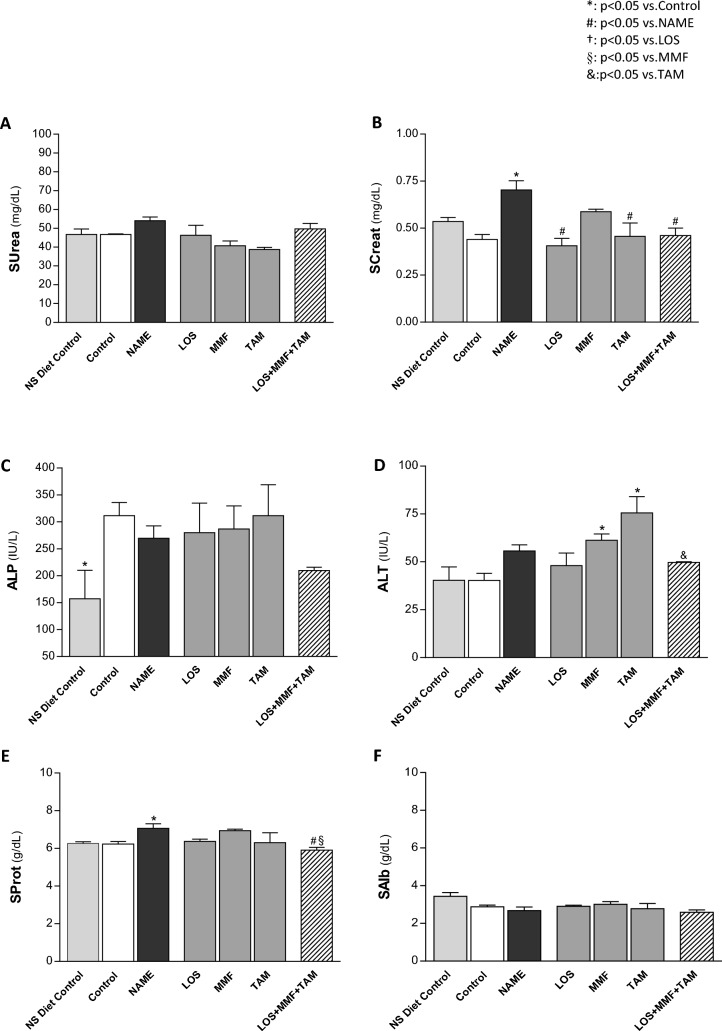
Renal and hepatic function tests: bar graph representation of (**A**) serum urea (SUrea, mg/dL), (**B**) creatinine (SCreat, mg/dL), (**C**) alkaline phosphatase (ALP, IU/L), (**D**) alanine transaminase (ALT, IU/L), (**E**) total protein (SProt, g/dL), and (**F**) albumin (SAlb, g/dL), concentrations in the groups: NS diet control, control, NAME, LOS, MMF, TAM and LOS + MMF + TAM. Results are presented as mean ± SEM. *p < 0.05 vs. Control, #: p < 0.05 vs. NAME, †: p < 0.05 vs. LOS, §: p < 0.05 vs. MMF, &: p < 0.05 vs. TAM.

Serum urea (SUrea) and creatinine (SCreat) concentrations were also determined in the experimental animals. The results can be seen in Fig. 2A and B. Untreated NAME rats exhibited only numerical increase in SUrea (54 ± 2 mg/dL), but a significant SCreat rising (0.70 ± 0.05 mg/dL), compared to Control animals (47 ± 1 and 0.44 ± 0.03 mg/dL, respectively). Creatinine retention was completely normalized by LOS (0.41 ± 0.04 mg/dL) and TAM (0.46 ± 0.07 mg/dL) monotherapies, as well as by the LOS + MMF + TAM association (0.46 ± 0.04 mg/dL).

Additionally, in order to verify the safety of LOS + MMF + TAM association, we performed hepatic function analysis through the biochemical dosage of proteins and enzymes (liver panel test) in the serum samples of all the studied animals. The results of these measures can be seen in the bar graphs of Fig. 2C–F. Wistar rats fed with HS diet presented high serum concentration of alkaline phosphatase (ALP), compared to the rats fed with conventional NS diet (311 ± 25 vs. 157 ± 24 IU/L). However, no significant differences were observed among the groups fed with HS diet, regardless of the treatment to which they were submitted. Of note, LOS + MMF + TAM animals exhibited the lower levels of ALP among HS fed rats (210 ± 6 IU/L). On the other hand, NAME rats presented numerically increased alanine transaminase (ALT) serum concentration, compared to Control animals (56 ± 3 vs. 40 ± 4 IU/L). Both MMF (61 ± 3 IU/L) and TAM (76 ± 9 IU/L) monotherapies promoted a further increase in this parameter, while LOS monotherapy (48 ± 7 IU/L) and LOS + MMF + TAM association did not change ALT concentration. Untreated NAME rats also exhibited increased serum protein (SProt) concentration, compared with Control rats (7.1 ± 0.2 vs. 6.2 ± 0.1 g/dL), which were normalized by LOS + MMF + TAM association (5.9 ± 0.2 g/dL).

### Treatment with LOS + MMF + TAM averted the development of glomerular injury

**Figure 3:**
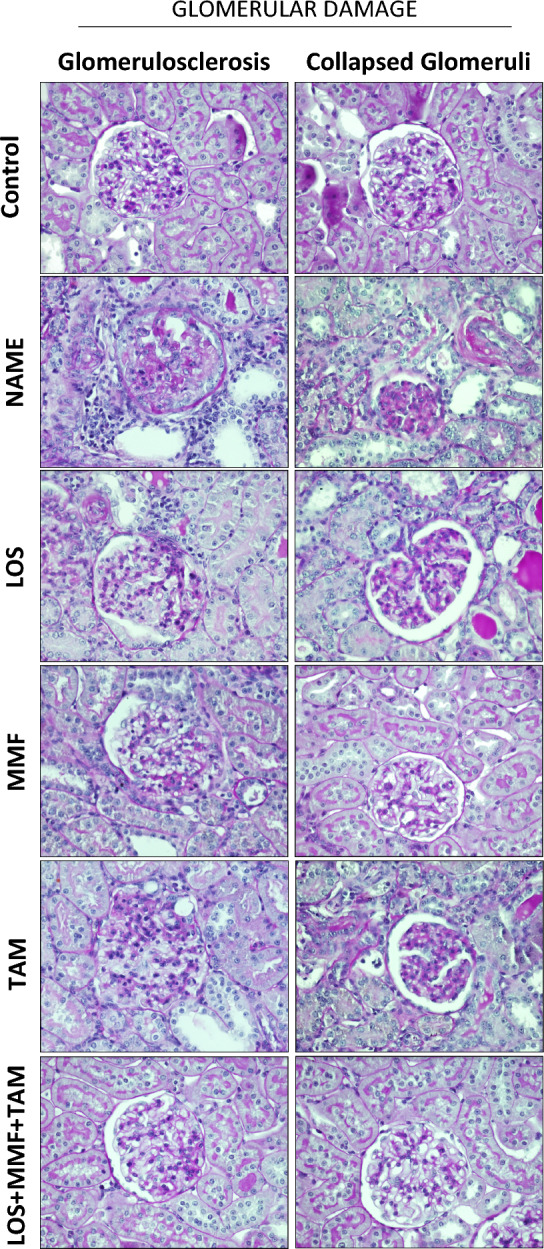
Representative micrographs of glomerulosclerosis (left) and collapsed glomeruli (right), accessed in the Control, NAME, LOS, MMF, TAM and LOS + MMF + TAM groups by PAS staining (400 × magnification).

**Figure 4:**
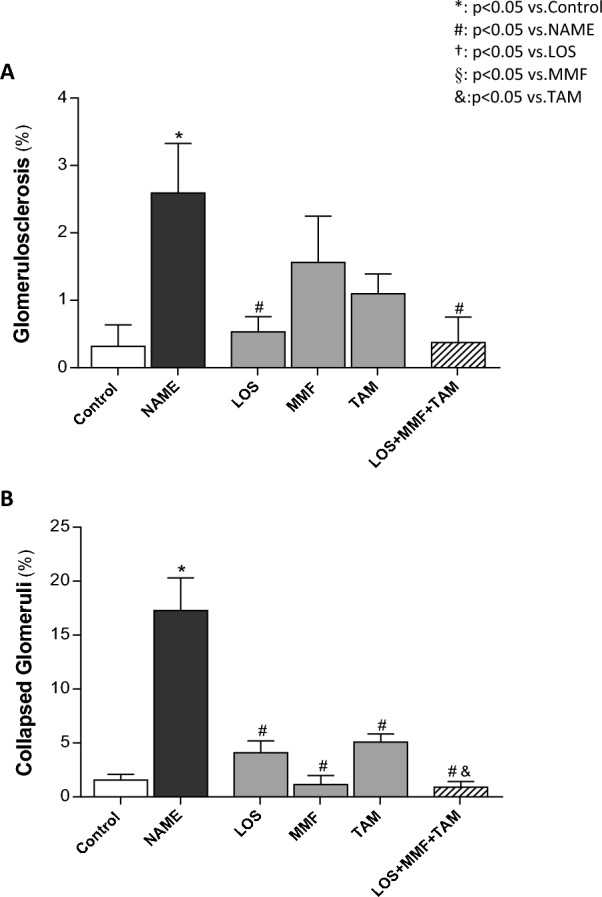
Bar graph representation of the percentage of glomerulosclerosis (**A**) and collapsed glomeruli (**B**) in the control, NAME, LOS, MMF, TAM and LOS + MMF + TAM groups. Results are presented as mean ± SEM. *p < 0.05 vs. Control, #: p < 0.05 vs. NAME, †: p < 0.05 vs. LOS, §: p < 0.05 vs. MMF, &: p < 0.05 vs. TAM.

Glomerular structural alterations, characterized by the development of glomerulosclerosis and by the presence of collapsed glomeruli, were accessed by PAS staining. Illustrative micrographs of samples of each experimental group are represented in Fig. 3. PAS staining highlights the extracellular matrix accumulation, as well as the thickening of basement membrane, in strong pink color. As expected, in Control rats we observed proportional distribution of cells and mesangial matrix in the glomerular tuft, accompanied by well-opened and visible capillary loops, absence of PAS-positive material accumulation foci, normal size of Bowman's space and regular thickness of the glomerular capsule. Untreated NAME rats exhibited prominent structural alterations, such as the emergence of collapsed glomeruli; characterized by a relative increase in Bowman’s space, thickening of the glomerular capsule, shrinkage and retraction of the glomerular tuft, occlusion of capillary loops, and greater PAS-positivity. It was also observed severe Glomerulosclerosis caused by ischemic injury in NAME rats receiving no therapeutic treatment. In this rats we observed the retraction and collapse of capillary tuft, which stains highly PAS-positive, surrounded by fibrotic tissue, which was PAS-negative. All the employed therapeutic schemes, partially averted glomerular lesions. Bar graphs of the respective quantitative analysis of the percentage of sclerotic and collapsed glomeruli are shown in Fig. 4. Accordingly, NAME animals shown significant glomerulosclerosis and glomerular collapse, compared to Control rats (2.6 ± 0.7 and 17.3 ± 3.0 vs. 0.3 ± 0.3 and 1.6 ± 0.5%, respectively). LOS, MMF and TAM monotherapies at least partially averted the development of both glomerulosclerosis and glomerular collapse in NAME rats (0.5 ± 0.2 and 4.1 ± 1.1%, 1.6 ± 0.7 and 1.2 ± 0.8%, 1.1 ± 0.3 and 5.1 ± 0.7%), and the association of LOS + MMF + TAM promoted further protection against these glomerular structural alterations (0.4 ± 0.3 and 0.9 ± 0.5%).

### Combined LOS + MMF + TAM ameliorated renal interstitial fibrosis

**Figure 5:**
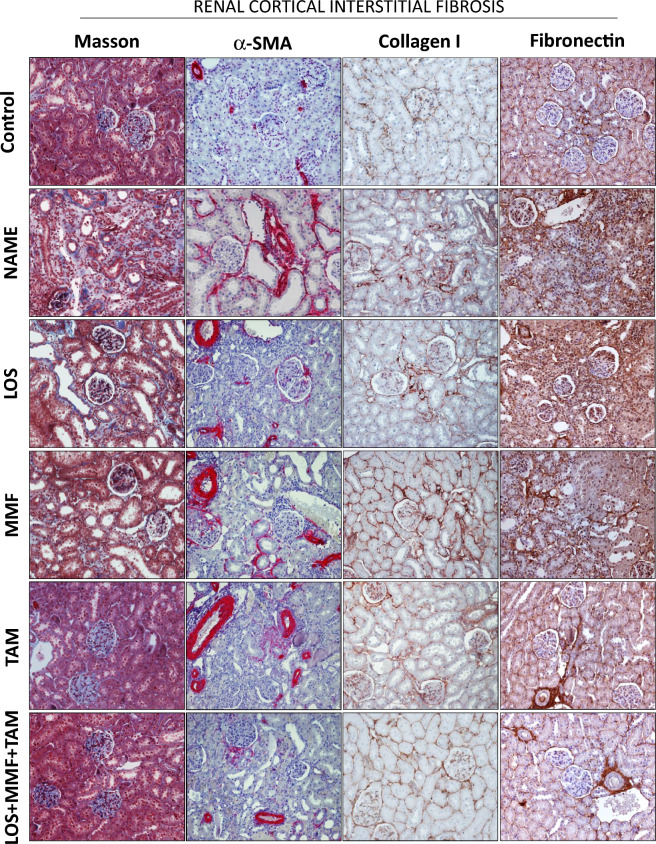
Representative micrographs of renal cortical Interstitial fibrosis, evaluated in Masson’s Trichrome stained slides, interstitial α-SMA, collagen I and fibronectin accumulation, accessed by immunohistochemistry in the Control, NAME, LOS, MMF, TAM and LOS + MMF + TAM groups (200× magnification).

**Figure 6:**
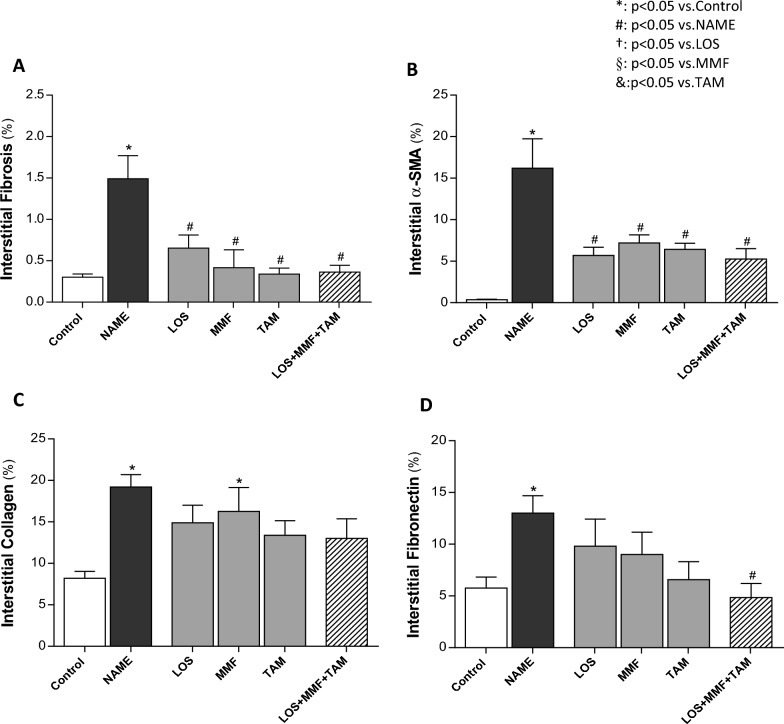
Bar graphs show quantification of percentage of cortical interstitial fibrosis (**A**), interstitial α-SMA accumulation (**B**), interstitial collagen I (**C**), and fibronectin (**D**), in the Control, NAME, LOS, MMF, TAM and LOS + MMF + TAM groups. Results are presented as mean ± SEM. *p < 0.05 vs. Control, #: p < 0.05 vs. NAME, †: p < 0.05 vs. LOS, §: p < 0.05 vs. MMF, &: p < 0.05 vs. TAM.

**Figure 7:**
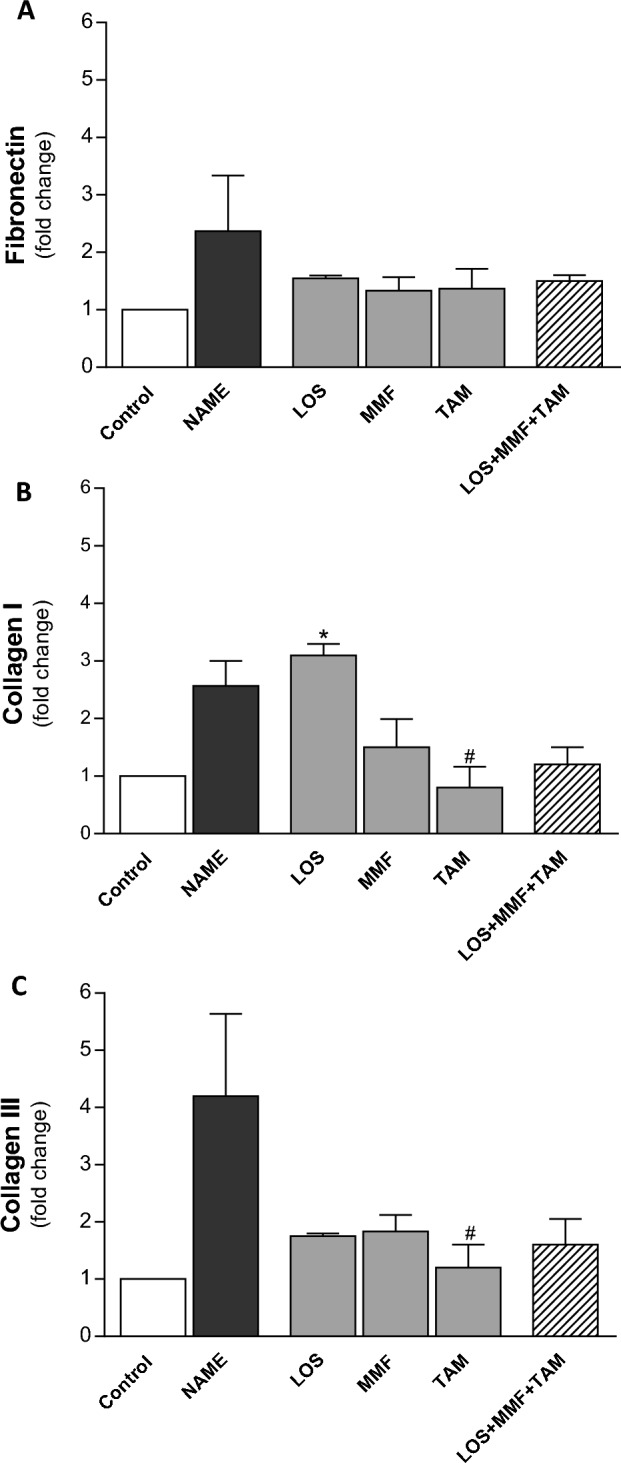
Quantitative RT-PCR of kidney samples from experimental animals, submitted to the different treatments. RNA expression of pro-fibrotic ECM proteins; fibronectin (**A**) collagen I (**B**) and collagen III (**C**), were presented as bar graphs. Results are presented as mean ± SEM. *p < 0.05 vs. Control, #: p < 0.05 vs. NAME, †: p < 0.05 vs. LOS, §: p < 0.05 vs. MMF, &: p < 0.05 vs. TAM.

As can be seen in the first column of illustrative microphotographs of Fig. 5, NAME animals exhibited severe renal interstitial fibrosis, evidenced in the Masson trichrome staining by the abundant positive areas, stained in blue. Dark blue marking can be seen around the renal tubules, as well as the accumulation of light blue stained ECM proteins occupying and expanding the renal interstitial area. Accordingly, in the second column of microphotographs, abundant interstitial α-smooth muscle actin (α-SMA) expression (in red), indicating the presence of myofibroblasts in the renal cortex, could be seen in NAME animals, compared to the Control. The overexpression of further ECM proteins related to renal fibrosis, such as collagen I (third column) and fibronectin (fourth column), both in brown, was also observed in NAME rats. Bar graphs of the quantitative analysis of these parameters were presented in Fig. 6. According to these quantifications, NAME animals exhibited significant renal fibrosis, evidenced by the positivity for Masson staining (1.7 ± 0.3 vs. 0.3 ± 0.1% in Control), as well as marked α-SMA accumulation (16.2 ± 3.5 vs. 0.4 ± 0.1% in Control), compared to Control rats. Both Masson positivity and the presence of interstitial myofibroblasts were equally limited by all the employed therapies, both monotherapies with LOS (0.8 ± 0.2 and 5.7 ± 1.0%), MMF (0.5 ± 0.4 and 7.2 ± 1.0%) and TAM (0.3 ± 0.1 and 6.4 ± 0.7%), and the association of drugs (0.4 ± 0.1 and 5.2 ± 1.2%). Interstitial collagen 1 and fibronectin percentages were also abnormally increased in untreated NAME rats compared to Control animals (21 ± 2 and 16 ± 1 vs. 11 ± 2 and 7 ± 1%, respectively). While the interstitial accumulation of collagen I was only subtly prevented by TAM monotherapy and the association of LOS + MMF + TAM (16 ± 2 and 17 ± 2%), interstitial fibronectin percentage was considerably averted by these treatments, especially by the combined therapy (11 ± 1 and 7 ± 2%). Further RT-qPCR analysis employed to determine the gene expression of collagens I and II and fibronectin in the renal tissue of experimental animals were performed. According to the representative bar graphs shown in Fig. 7, untreated NAME rats exhibited an increase of 2.5 fold in both fibronectin and collagen I expressions, and an increase of 4.2 fold in collagen III gene expression, compared to the Control animals. TAM in monotherapy or in association with LOS and MMF reduced the expression of this three profibrotic genes.

### LOS + MMF + TAM association prevented renal inflammation

**Figure 8:**
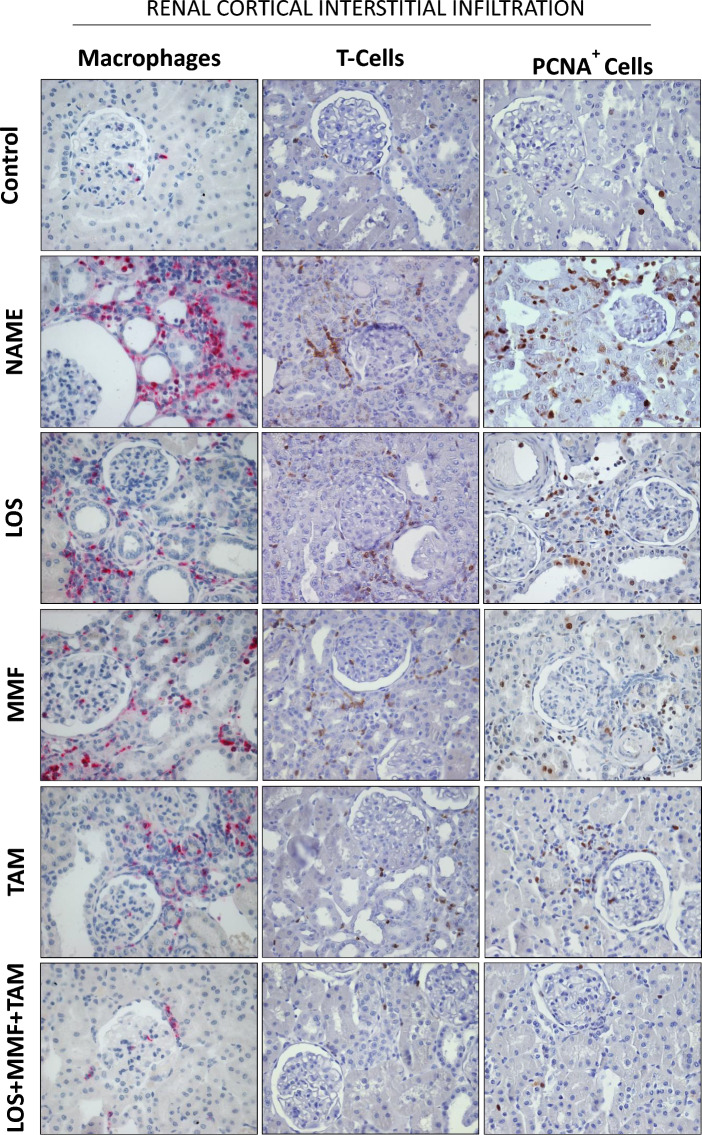
Representative micrographs of renal cortical interstitial infiltration of macrophages, lymphocytes (T-Cells) and PCNA^+^ cells, accessed by immunohistochemistry in the Control, NAME, LOS, MMF, TAM and LOS + MMF + TAM groups (400× magnification).

**Figure 9:**
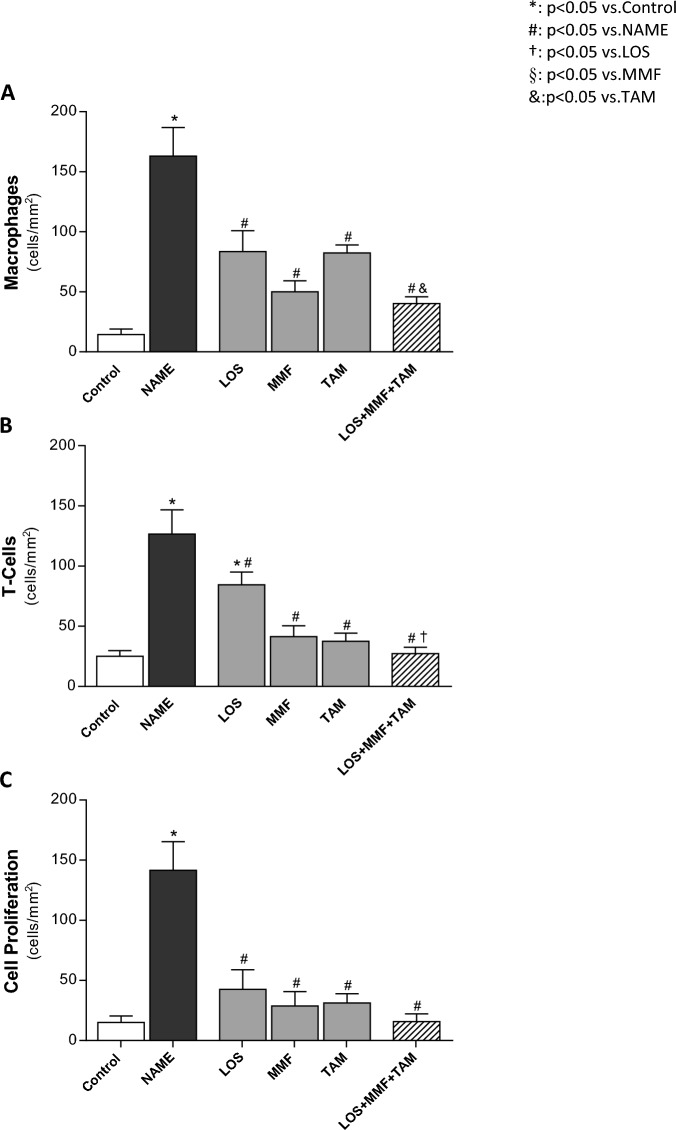
Bar graphs show interstitial infiltration of macrophage (**A**), T-Cell (**B**) and PCNA^+^ cells (**C**) in the control, NAME, LOS, MMF, TAM and LOS + MMF + TAM groups. Results are presented as mean ± SEM. *p < 0.05 vs. Control, #: p < 0.05 vs. NAME, †: p < 0.05 vs. LOS, §: p < 0.05 vs. MMF, &: p < 0.05 vs. TAM.

Furthermore, illustrative microphotographs of immunohistochemistry for the detection of renal infiltration by macrophages (cells in red) and T-cells (in brown) and for the evaluation of renal interstitial cell proliferation (PCNA-positive cells stained in brown), in renal sections of animals from each experimental group, can be seen in Fig. 8. Immunohistochemistry analysis demonstrated that untreated rats submitted to the CKD model induced by L-NAME administration presented marked renal inflammation, characterized by intense cortical infiltration by macrophages and T-lymphocytes, and increased interstitial cell proliferation, evidenced by the presence of interstitial PCNA-positive cells. The inflammatory cells were detected mainly in the renal interstitium, but were also observed infiltrating glomeruli and in the perivascular area. According to the quantification of these cells, presented in Fig. 9, untreated NAME rats exhibited significant interstitial macrophage (163 ± 24 cell/mm^2^) and T-cell (127 ± 20 cell/mm^2^) infiltration, compared to the Control (14 ± 5 and 25 ± 5 cell/mm^2^). LOS, MMF and TAM monotherapies statistically reduced these cells (84 ± 17 and 84 ± 11 cell/mm^2^, 50 ± 9 and 42 ± 9 cell/mm^2^ and 82 ± 7 and 38 ± 7 cell/mm^2^, respectively). LOS + MMF + TAM association achieved the numerically lowest values of both macrophage and lymphocyte interstitial infiltration cells (40 ± 6 and 27 ± 5 cell/mm^2^). Similar results were obtained regarding renal cortical cells proliferation. NAME group presented significant increase in PCNA^+^ interstitial cells, compared to Control (142 ± 24 vs. 15 ± 5 cell/mm^2^). All the employed monotherapies reduced cell proliferation in this experimental nephrosclerosis model (LOS: 43 ± 16, MMF: 29 ± 12 and TAM: 31 ± 8 cell/mm^2^), and once more, the lowest number of positive cells were observed with the association of LOS + MMF + TAM (16 ± 6 cell/mm^2^), in which group the interstitial cell proliferation rate was comparable to the observed in the Control.

### TAM in monotherapy or associated to LOS effectively inhibited fibroblasts activation and ECM overproduction in cultured NRK-49F cells

**Figure 10:**
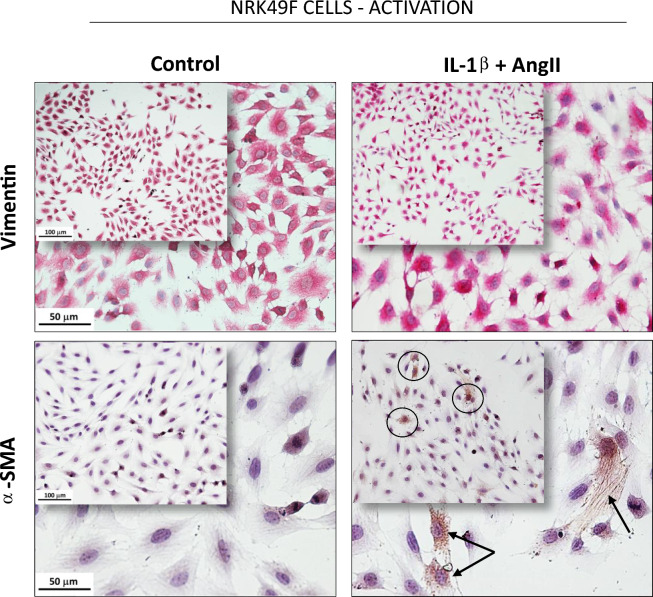
Representative micrographs of immunocytochemistry of untreated (Control) and IL-1β + AngII-stimulated NRK49F cells, stained for both a constitutive (vimentin) and a fibroblast activation-related (α-SMA) protein.

In order to establish an in vitro model of activated fibroblasts, which may mimic the subpopulation of renal fibroblasts of our in vivo experimental nephrosclerosis model, we stimulate rat renal immortalized fibroblasts from a commercially available cell line (NRK-49F) with IL-1β + AngII. As shown in Fig. 10, after this stimulus, NRK-49F continued to express vimentin, a cytoskeleton type III intermediate filament, constitutively present in both fibroblasts and myofibroblasts, but also began to express α-SMA, indicating the effective fibroblast activation and differentiation of part of these cells to myofibroblast.

**Figure 11:**
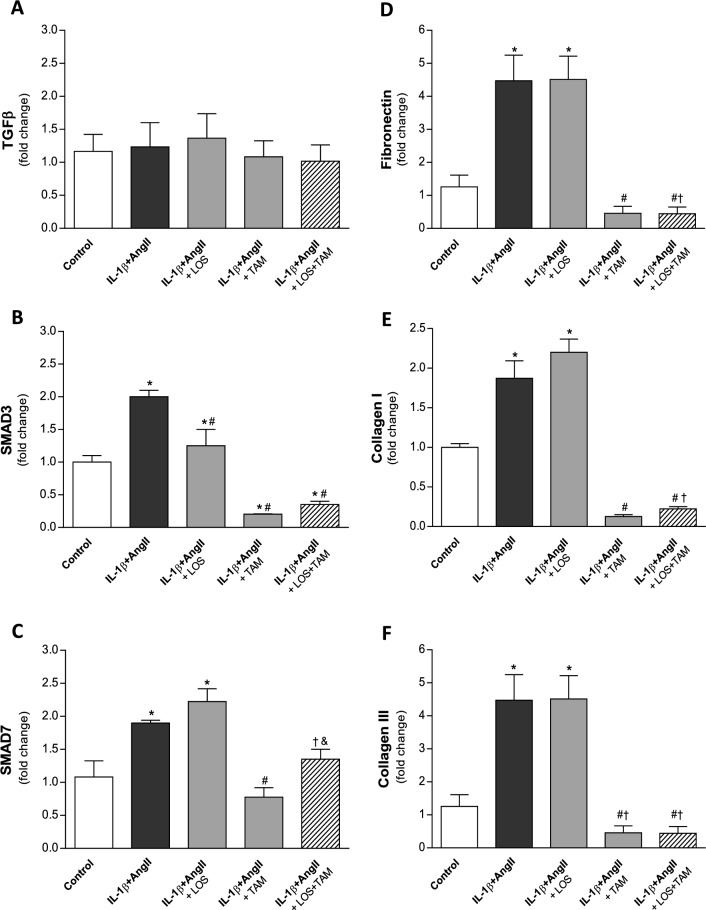
Quantitative RT-PCR of cultured NRK49F cells, submitted to the different treatments. RNA expression of pro and anti-fibrotic signaling factors; TGFβ (**A**), SMAD3 (**B**) and SMAD7 (**C**), as well as for the ECM proteins; collagen I (**D**) collagen III (**E**) and fibronectin (**F**), were presented as bar graphs. Results are presented as mean ± SEM. *: p < 0.05 vs. Control, #: p < 0.05 vs. IL-1β + AngII, †: p < 0.05 vs. IL-1β + AngII + LOS, §: p < 0.05 vs. IL-1β + AngII + TAM.

**Figure 12:**
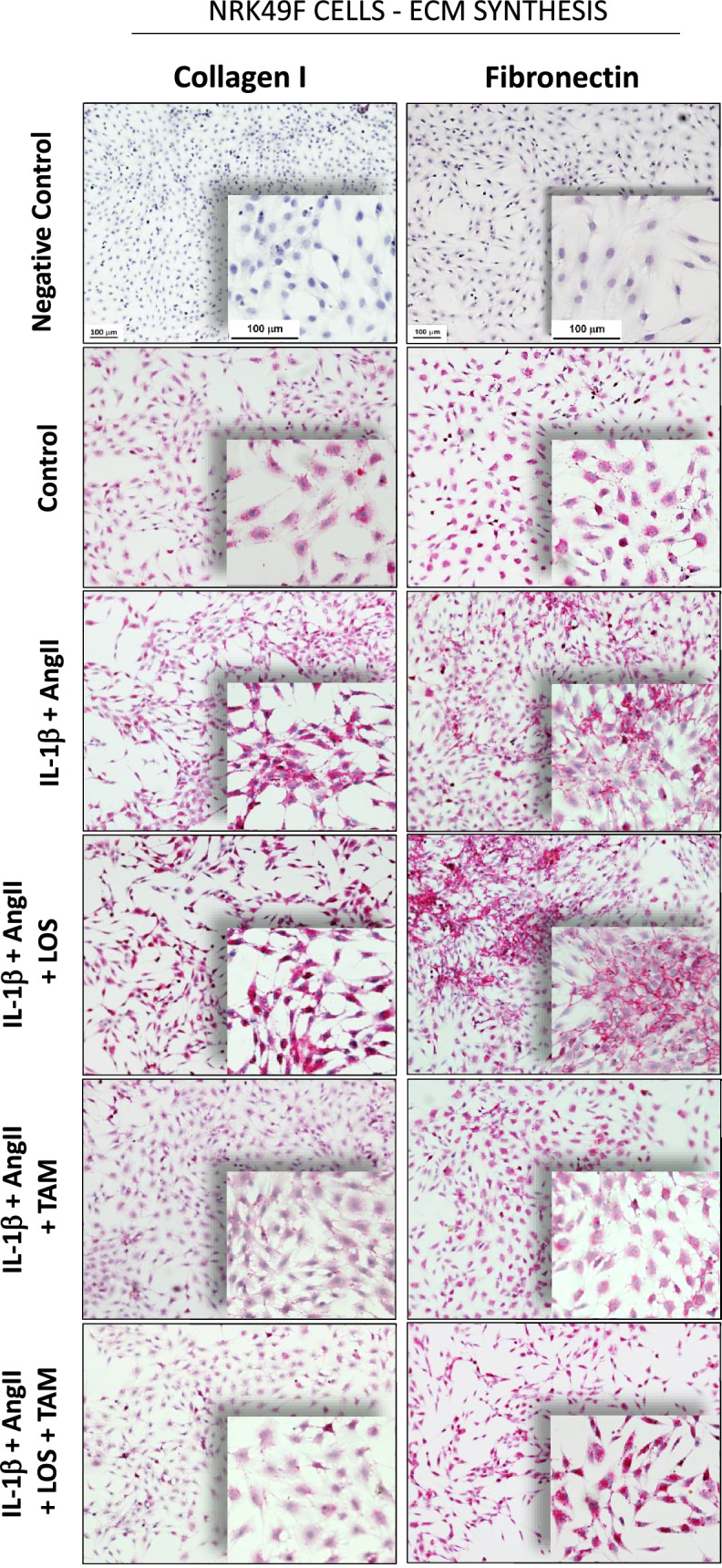
Representative micrographs of immunocytochemistry of NRK49F cells, stained for collagen I and fibronectin.

The results of RT-qPCR analysis of gene expression of pro and antifibrotic factors in NRK-49F cells are shown in Fig. 11. IL-1β + AngII stimulus upregulated the expression of SAMD3 and SMAD7, as well as the expression of fibronectin, collagen I and collagen III in NRK-49F cells. While LOS treatment only reverted partially the overexpression of SMAD3, both TAM and associated LOS + TAM significantly normalized the expression of fibronectin, collagen I and collagen III, and reduced SMAD3 expression to levels lower than the observed in Control NRK49F cells. Additional illustrative immunocytochemistry for fibronectin and collagen I performed in cultured NRK-49F cells are shown in Fig. 12, in which is it possible to verify that, untreated IL-1β + AngII-stimulated NRK-49F and LOS-treated cells exhibited exuberant positivity for fibronectin, suggesting fibronectin assembly and ECM overproduction, compared with the unstimulated NRK-49F or to the TAM and LOS + TAM-treated cells.

## Discussion

In the present study we investigated the potential renoprotective effects of the therapeutic association of LOS + MMF + TAM on an experimental model of hypertensive nephrosclerosis, based on the chronic inhibition of NO synthesis, induced by L-NAME administration. The main aim of our research was to verify whether the combination of TAM, a selective estrogen receptor modulator, recommended for the treatment of positive estrogen receptor (ER+) breast cancer, to the currently employed conservative CKD treatment, here represented by RAAS blockade and immunosuppression, would promote additional anti-inflammatory and/or antifibrotic beneficial effects, when compared to the respective monotherapies.

Corroborating previous data, rats submitted to the L-NAME model of CKD developed severe hypertension, probably caused by glomerular and systemic vasoconstriction due to the lack of physiological vasodilatory effects of NO. Systemic hypertension and CKD are closely related conditions with an intricate cause/effect relationship. The decline of kidney function usually leads to high blood pressure, due to both a decreased ability of the kidneys to remove salt from the bloodstream, and an increased release of renal vasoconstrictive hormones. On the other hand, systemic hypertension sustained for long periods leads to the damage of multiple target organs, including the kidneys^7,8^. Hypertensive nephrosclerosis is one of the main causes of end-stage renal failure. High blood pressure also contributes to the aggravation of CKD, regardless of its etiology^1^. Accordingly, the clinical management of hypertension is currently one of the most employed strategies to control CKD progression^7,8^. According to our results, systemic hypertension induced by L-NAME administration was only partially reduced by the monotherapies with both LOS or MMF, and equally by the association of LOS + MMF + TAM. There was no synergistic effect of the combination of drugs as regards lowering blood pressure. The poor hemodynamic effect of therapeutic schemes may have limited the potential effects of the therapeutic association on the maintenance of renal function.

Along with the hypertension, NAME animals also exhibited a markedly increased urine albumin excretion rate, a clear evidence of renal impairment. Because of its strong predictive power for cardiovascular and renal events, albuminuria is one of the most important biomarkers of CKD progression, particularly in patients with hypertension or diabetes mellitus. Moreover, the reduction of albuminuria is the most important goal to prevent the progression of kidney disease in CKD patients. Usually, in this regard, significant benefits are achieved by the therapeutic treatment with RAAS inhibitors. According to our results, although all the tested monotherapies significantly limited the development of albuminuria in NAME animals, the antiproteinuric effect obtained with the combined treatment was noteworthy. Is spite of the severe sustained hypertension, LOS + MMF + TAM association completely averted the development of albuminuria in this CKD model.

The increased urinary albumin excretion is directly related to the disruption of one or more components of the glomerular filtration barrier, and with glomerular structural damage, generally caused or worsened by renal inflammation. Histological glomerular alterations are a common feature in most human and experimental nephropathies. Accordingly, severe glomerulosclerosis and glomerular collapse were observed in untreated NAME animals, and the association of LOS + MMF + TAM significantly prevented the development of glomerular histological damage, probably reflecting the effects of LOS (for glomerulosclerosis) and MMF (for collapsed glomeruli), thus evidencing that TAM did not exerted antagonism, blockade or inhibition upon the pharmacological effects of both LOS and MMF, and did not diminish the renoprotective effects observed with these drugs.

Kidney infiltration by inflammatory leukocytes has been demonstrated in a variety of non-immune mediated nephropathies, such as the hypertensive nephrosclerosis^4,19,23,24^. The recruitment of circulating monocytes, as well as the activation of resident renal macrophages often correlates positively with the worsening of renal function loss, in both human and experimental CKD^2^. Accordingly, in the present study, NAME animals showed exuberant renal inflammation, characterized by inordinate tubulointerstitial cell proliferation and massive infiltration of kidneys by both macrophages and lymphocytes. Surprisingly, similarly to the observed with LOS and MMF monotherapies, expected to exert inhibitory effects on macrophage and lymphocyte renal infiltration, as well as on interstitial proliferation rate, TAM monotherapy also exhibited independent significant anti-inflammatory proprieties. Therefore, the association of LOS + MMF + TAM seems to combine different mechanisms of action to abrogate renal inflammation. Inhibition of macrophage activity by TAM treatment has been demonstrated in both in vitro and in vivo studies, in which Tamoxifen promoted significant reduction in the transcription of important cell surface receptors, such as the fatty acid-binding proteins (FABPs) and the scavenger receptor class B member 3 (SCARB3/CD36), which are involved in the monocyte/macrophage activation processes, and play a pivotal role in foam cell formation and in the development of atherosclerosis^25^. The anti-proliferative effects of RAAS blockade, associated with the reduction of IL1, IL6 and IL10 macrophage release, possibly achieved with the MMF treatment, may have boosted the anti-inflammatory effects of TAM. A synergetic effect among the tested drugs could also be plausible in this case. However, additional studies focused on the intracellular mechanisms of action of each employed drug and in the possible chemical interactions among them should be carried out to speculate this hypothesis.

Along with renal inflammation, the overproduction of ECM and the renal interstitial collagen accumulation are important histological features, commonly related to the worsening of CKD. In the present study renal cortical interstitial fibrosis, evidenced by the high percentage of Masson^+^ interstitial staining, myofibroblasts infiltration, as well as collagen I and fibronectin interstitial deposition, accompanied the progression of hypertensive nephrosclerosis in untreated NAME rats. All the tested therapies were effective in preventing renal fibrosis and α-SMA accumulation, while only TAM and the association of LOS + MMF + TAM significantly reduced collagen I and fibronectin accumulation in this CKD model, suggesting that TAM promoted additional antifibrotic effect to the therapeutic scheme, with no impairment of renoprotective action of LOS and MMF, when associated to these drugs. The suppressive effects of tamoxifen on fibrogenesis were first described in the early nineties, when Clark and collaborators described the drug to be effective and safe in the treatment of two patients with severe retroperitoneal fibrosis. Its effectiveness for the treatment of encapsulating peritoneal sclerosis, where than demonstrated, 8 years later, by Allaria and co-authors. Based on these observations, Dellê and collaborators, from our research group, showed for the first time that TAM could exert protective effects on experimental progressive chronic kidney disease, in 2003.^18,26,27^. More recently, TAM was described to exert important antifibrotic effects in the experimental model of unilateral ureteral obstruction (UUO) in mice. Similarly, to the observed in the present study, TAM treatment reduced the production and deposition of ECM proteins in UUO kidneys, as well as the renal deposition of fibronectin and collagen^28,29^. Although the exact mechanisms involved in the anti-inflammatory and antifibrotic effects of TAM are still poorly known, it exerts undoubted suppressive effects on fibroblast proliferation, activation and ECM secretion, evidenced in our in vitro results, which corroborate the current literature^30^. Since MMF, employed as an immunosuppressive drug in the in vivo protocol, is in fact a prodrug, which must be ingested and then metabolized into the pharmacologically active drug (mycophenolic acid), we were not able to perform in vitro studies with the full LOS + MMF + TAM association. However, we combined LOS to TAM is our analysis and clearly demonstrated that LOS did not impaired the suppressive effects of TAM on cultured fibroblasts, thus corroborating the idea that this drug combination may be safe and effective.

In summary, although further studies employing different CKD models are still required to confirm the efficacy and safety of the association of LOS + MMF + TAM, in the present paper we provided strong evidence that this therapeutic scheme can be potentially useful to slow the progression of chronic nephropathy, since it lowered systolic blood pressure, prevented albuminuria, glomerular structural damage, and renal inflammation and promoted additional antifibrotic effect to the traditional conservative treatment of CKD, in the NAME model of hypertensive nephrosclerosis. In conclusion, our pre-clinical observations suggested that the association of TAM to the conservative treatment of CKD, employing LOS and MMF, was safe and promoted additional renoprotective, anti-inflammatory and antifibrotic effect in a model of hypertensive nephrosclerosis in rats.

## Material and methods

### In vivo experimental groups and protocol

The present experimental protocol was approved by the local Research Ethics Committee (Comissão de Ética para Análise de Projetos de Pesquisa—CAPPesq) and was developed in strict conformity with the international standards for care and manipulation of laboratory animals.

Male Wistar rats aged between 7 and 8 weeks were kept under controlled temperature (23 ± 1 °C), on a 12/12 h’ light/dark cycle with ad libitum access to tap water and HS diet (3.2% Na, Nuvital, Brazil). After 2 weeks of adaptation to HS diet, 60 of these animals were submitted to the NAME experimental model: As previously described, these model of hypertensive nephrosclerosis was induced by the chronic inhibition of endogenous NO, thus stimulating peripheral vasoconstriction^4^. NO synthesis blockage was obtained by oral daily administration of 70 mg/kg/day of Nω-Nitro-l-arginine methyl ester hydrochloride (L-NAME, Sigma-Aldrich #N5751), a L-arginine analogue, diluted on drinking water, associated to the HS diet.

NAME rats were divided among the following 5 groups: **NAME:** Animals submitted to the NAME model and keep untreated; **LOS:** NAME animals treated with 50 mg/Kg/day of losartan (LOS, Losartana Potássica 50mg, Medley) diluted in drinking water; **MMF:** NAME rats treated with 10 mg/Kg/day of Micofenolate Mofetil (MMF, Micofenolato de Mofetila, 500 mg, Accord-Intas Pharmaceuticals), administered daily by oral gavage; **TAM:** NAME animals receiving 10 mg/Kg/day of Tamoxifen (TAM, Citrato de Tamoxifeno, 20 mg, Sandoz-Novartis) by oral gavage, and **LOS + MMF + TAM:** NAME rats treated with LOS, MMF and TAM simultaneously. Five additional animals received only HS and were used as **Control**. An illustrative flow-chart depicting the study design and groups can be seen in Supplemental Material section, on Supplementary Fig. 1A. Additionally, further animals were kept with normal sodium diet (standard rodent chow, Nuvital, Brazil), and used as NS Diet Control for clinical and biochemical analysis.

All groups were followed for 30 days. Body weight was monitored weekly and at the end of this period, systolic blood pressure was evaluated by the tail-cuff pressure method, using a noninvasive system (Visitech Systems, Apex, NC). 24-h urinary albumin excretion rate (24 h-UAE) was analyzed by radial immunodiffusion, as described elsewhere^4,20^. Animals were anesthetized with an intraperitoneal (IP) injection of 60 mg/kg of sodium pentobarbital and submitted to a ventral laparotomy. Blood samples were collected directly from the abdominal aorta, for renal and hepatic function analyses, followed by euthanasia through overdose of sodium pentobarbital, 80 mg/kg IP. The left kidney was removed and processed for histological and immunohistochemical analysis, while the right kidney was cut into small fragments and rapidly frozen in liquid nitrogen for PCR analysis.

### Renal and hepatic function

Blood samples were centrifuged at 2000 rpm for 15 min, at room temperature for serum obtaining. Renal and hepatic function analyses were performed in serum samples of the animals of each experimental group: Blood Urea Nitrogen (SUrea) and serum creatinine concentration (SCreat) were assessed by colorimetric methods (Labtest Diagnóstica do Brasil UREIA CE #37 and CREATININA #35, respectively). Total serum protein (SProt), albumin (SAlb), alanine aminotransferase (ALT) T and alkaline phosphatase (ALK) concentrations were determined using specific colorimetric assay kits (Labtest Diagnóstica do Brasil, #99-1, #19-1, #108-4 and #79-4, respectively).

### Histological analysis

As described elsewhere^4^, kidneys obtained from total nephrectomy were cut in two midcoronal renal slices and pre-fixed with Duboscq-Brazil for 30 min, followed by 24-h post-fixation in buffered 4% formaldehyde. Tissue samples were embedded in paraffin, through conventional techniques, renal tissue sections of 4-μm thickness were obtained and submitted to histological analysis for the assessment of glomerular and tubulointerstitial alterations.

The percentage of glomerulosclerosis and collapsed glomeruli were evaluated in periodic Acid-Schiff (PAS) staining samples, through the analysis of at least 50 randomly sampled glomerular tuft profiles per rat. The criteria used to define sclerotic glomeruli was the presence of segmental hyalinosis lesions, usually with adhesion to Bowman’s capsule. Collapsed glomeruli were defined by their reduced size, wrinkling basement membrane and collapsed capillary loops. Interstitial fibrosis was quantitatively evaluated in Masson-stained sections by a point counting technique^4,21^.

### Immunohistochemical analysis

Immunohistochemistry (IHC) assays were performed to identify interstitial macrophage and T-cell infiltration in renal sections, as well as to evaluate tubulointerstitial cell proliferation and to quantify the percentage of tubulointerstitial area occupied by α-smooth muscle actin (α-SMA), possibly indicating the presence of myofibroblasts in the renal cortex^2^, collagen I and fibronectin. A mouse monoclonal anti-ED1 antibody (Serotec, Oxford, UK) was used to identify macrophages through the APAAP (alkaline phosphatase anti-alkaline phosphatase) technique. Monoclonal mouse anti-CD3 (Dako, Glostrup, Denmark) and anti-α-SMA (Sigma Chemical CO, St. Louis, USA) antibodies were used, to identify T-cells and myofibroblast, respectively, through a streptavidin–biotin-alkaline phosphatase (Strep-AP) IHC technique. In both APAAP and Strep-AP techniques, the reactions were developed with a fast-red dye solution. Tubulointerstitial cell proliferation was detected by a monoclonal mouse anti-PCNA antibody (Dako, Glostrup, Denmark), while collagen I and fibronectin positivity were detected with polyclonal anti-collagen I (Rockland Immunochemicals, Inc., NY, USA) and anti-fibronectin (Sigma Chemical CO, St. Louis, USA) primary antibodies, using a streptavidin-biotin-horseradish peroxidase (Strep-HRP) IHC technique. Samples were developed with a DAB dye solution.

### In vitro experiments

In order to verify the specific antifibrotic activity of TAM alone, and also to investigate if this activity would be somehow inhibited or impaired by the association with other drugs, we performed cell culture experiments using a rat renal fibroblast cell line (NRK-49F; American Type Culture Collection, Manassas, VA). For this purpose, 1 × 10^5^ NRK-49F cells were cultured under 37 °C and 5% CO_2_ in plastic culture plates with Dulbecco’s Modified Eagle Medium (DMEM-Low glucose, Invitrogen, USA) containing 5% inactivated fetal bovine serum (FBS; Gibco, Carlsbad, MO, USA), 100 units/mL penicillin, and 100 mg/mL streptomycin antibiotic solution (Gibco). Once cells reached 80% of confluence, the culture medium was replaced by DMEM-Low with 100 units/mL penicillin, and 100 mg/mL streptomycin antibiotic solution, plus the specific stimuli, as follows; Control, NRK-49 cells receiving no additional stimuli or treatment diluted in the culture medium, IL-1β + AngII, NRK-49 cells whose culture medium was supplemented with 400 pg/mL of recombinant human IL-1β (PeproTech, Cranbury, NJ, USA) and 1 × 10^−7^ M human Angiotensin II acetate (Sigma), LOS, IL-1β + AngII cells whose culture medium was further supplemented with 10 µM of Losartan, TAM, IL-1β + AngII cells whose culture medium was further supplemented with 5 µM of Tamoxifen citrate (Sigma) and LOS + TAM, NRK-49 cells receiving all the above mentioned supplements. Cells were kept under the described treatments for 24 h. An illustrative flow-chart of in vitro experiments can be seen in Supplemental Material section, on Supplementary Fig. 1B.

### Immunocytochemistry

Immunocytochemistry (ICC) assays were performed to characterize the constitutive expression of vimentin in NRK-49 cells, using a mouse monoclonal anti-vimentin primary antibody (Sigma Chemical CO, St. Louis, USA). The activation of fibroblasts after IL-1β + AngII stimulus, was evaluated through the positivity of these cells for α-SMA, with a mouse monoclonal anti-α-SMA antibody (Sigma Chemical CO, St. Louis, USA). Moreover, ICC was also employed to analyze the expression of collagen I and fibronectin in NRK-49 cells submitted to the different treatments, employing, respectively, the rabbit polyclonal anti-collagen I (Rockland Immunochemicals, Inc., NY, USA) and anti-fibronectin (Sigma Chemical CO, St. Louis, USA) primary antibodies. Vimentin, collagen I and fibronectin ICC were performed through a streptavidin-biotin-alkaline phosphatase (Strep-AP) technique. Reactions were developed with a fast-red dye solution. α-SMA was detected through a streptavidin-biotin-horseradish peroxidase (Strep-HRP) ICC technique, developed with a DAB dye solution.

### Real time RT-PCR

Quantitative real-time polymerase chain reaction (PCR) of renal samples of experimental animals, as well as from cultured NRK-49F cells was performed to measure the relative gene expression of TGFβ, SMAD3, SMAD7, Collagen type I, collagen type III and Fibronectin, using Actinβ as a housekeeping control, as previously described. Total RNA extraction was carried out with RNeasy Plus Kit (Qiagen, MD, EUA), following the instructions of the manufacturer. Reverse transcription (RT) was performed with M-MLV enzyme kit (Promega) and qPCR was conducted with the Syber GreenER qPCR Super Mix Universal (Invitrogen), in the StepOne Plus equipment (Applied Biosystetems—Life Technologies). Quantitative comparisons were obtained using the ΔΔCT method (Applied Biosystems, Singapore, Singapore). Primer sequences for amplifying target genes were: Tgfb1 NM_021578.2, left primer: GCTGAACCAAGGAGACGGAA, right primer: CATGAGGAGCAGGAAGGGTC, Smad3 NM_013095.3 left primer: GAGACATTCCACGCTTCACA, right primer: AAAGACCTCCCCTCCAATGT, Smad7 NM_030858.2 left primer: TCTCCCCCTCCTCCTTACTC, right primer: CAGGCTCCAGAAGAAGTTGG, Coll1a1 NM_053304.1 left primer: AGCTGGTGCTAAGGGTGAAG, right primer: GCAATACCAGGAGCACCATT, Coll3a1 NM_053304.1 left primer: AGCTGGTGCTAAGGGTGAAG, right primer: GCAATACCAGGAGCACCATT, Fn1 NM_019143.2 left primer CTCCCGGAACAGATGCAATG, right primer ATCCAGCTGAAGCACTCTGT and Actb NM_031144.3 left primer: AGGGAAATCGTGCGTGACAT, right primer: CCATACCCAGGAAGGAAGGC.

### Statistical analysis

Results were presented as mean ± SEM. Differences among all groups were analyzed by one-way ANOVA with Dunnet’s multiple comparison post-test. Means were considered statistically different when p < 0.05^22^. All statistical analyses were realized using the Graph-Pad Prism™ 5.01 software.

### Ethical approval

The present experimental protocol was approved by the local Research Ethics Committee (Comissão de Ética para Análise de Projetos de Pesquisa—CAPPesq) and was developed in strict conformity with the international standards for care and manipulation of laboratory animals.

### Statement of adherence to The ARRIVE Guidelines

The present experimental study was reported in accordance with The ARRIVE guidelines 2.0, https://doi.org/10.1371/journal.pbio.3000411, following the ARRIVE Essential 10 requirements.

### Supplementary Information

Supplementary Information.

## Data Availability

All data generated in the present study are included in this published article.
