# Supplementary material for: Tamoxifen associated to the conservative CKD treatment promoted additional antifibrotic effects on experimental hypertensive nephrosclerosis
Source: Sci Rep. 2023 Aug 26;13:13985. doi: 10.1038/s41598-023-39299-9 (PMC10460450; doi:10.1038/s41598-023-39299-9)
Supplement: Supplementary file 1 — Supplementary Information. [file 41598_2023_39299_MOESM1_ESM.docx]

**A**

**Supplementary Figure 1**


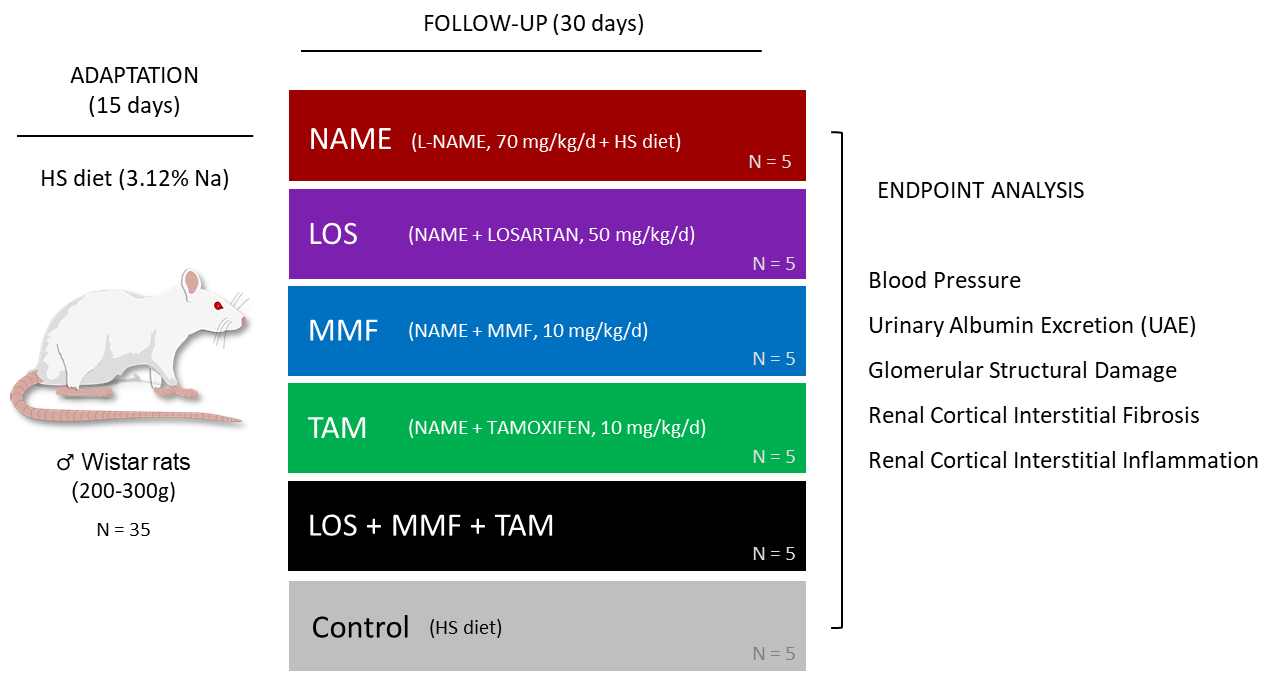


HS diet (3.2% Na)

**B**


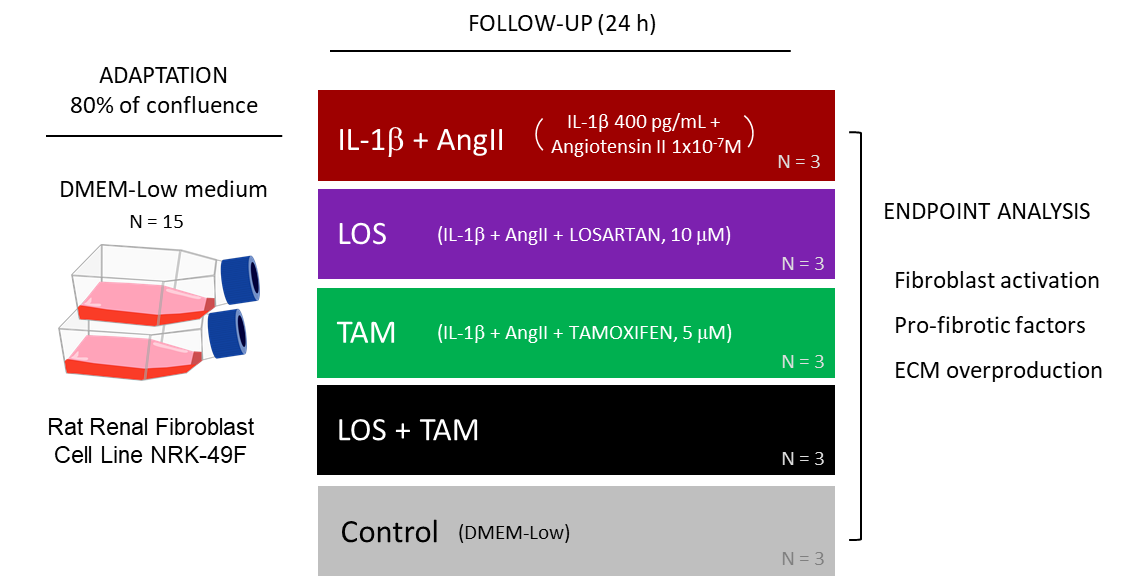


**Supplementary Figure 1.** Illustrative flow-chart depicting the *in vivo* (A) and *in vitro* (B) study protocols

**Supplementary Figure 2**

**Supplementary Figure 2.** Scatter-plot graph of individual Systolic Blood Pressure data (SBP, mmHg) in the NS Diet Control, Control, NAME, LOS, MMF, TAM and LOS+MMF+TAM groups. Results are presented as mean ± SEM. *p<0.05 vs. Control, #: p<0.05 vs. NAME, †: p<0.05 vs. LOS, §: p<0.05 vs. MMF, &: p<0.05 vs. TAM.

Data are presented as Mean±SE. p<0.05 vs. a)NS Diet Control, b)Control, c)NAME, d)LOS, e)MMF, f)TAM, g)LOS+MMF, h)LOS+TAM, i)MMF+TAM. Body Weight (BW), Systolic Blood Pressure (SBP), Urinary Volume (UV), Urinary Protein Excretion (UPE), Urinary Albumin Excretion (UAE), Serum Urea (SUREA), Serum Creatinine (SCREAT), Serum Alanine Transaminase (ALT), Serum Alkaline Phosphatase (ALK), Serum Protein Concentration (SPROT), Serum Albumin Concentration (SALB), Interstitial Fibrosis (IntFib).

**Supplementary Table 1.** General results of all monotherapy and combined experimental groups
